# Immuno-Diagnostic Interest in Monitoring CD16+CD56+ (Natural Killer) Cells and CD19+CD45+ (B Lymphocytes) in Individuals Newly Diagnosed with HIV in a Tertiary Care Center

**DOI:** 10.3390/jcm13041154

**Published:** 2024-02-18

**Authors:** Jamil Al-Mughales

**Affiliations:** 1 Department of Clinical Microbiology and Immunology, Faculty of Medicine, King Abdulaziz University, Jeddah 21589, Saudi Arabia; jmughales@kau.edu.sa; 2 Department of Clinical Laboratories, Diagnostic Immunology Division, King Abdulaziz University Hospital, Jeddah 23623, Saudi Arabia

**Keywords:** CD16+ (natural killer), CD19+ (phenotype), HIV

## Abstract

Background/Objective: Monitoring multiple cellular markers of immune cells may provide a more accurate evaluation of the immune status of people living with human immunodeficiency virus (PLHIV). This study assessed the value of CD16+CD56+ cells (NK cells) and CD19+ lymphocytes (B cells) phenotyping in indicating viral load, AIDS status, and treatment efficacy. Method: A retrospective, laboratory-based study was conducted at the Diagnostic immunology division of a referral tertiary hospital. It involved 82 newly diagnosed HIV patients treated between 2009–2016. We explored three objectives: (1) the paired change in CD16+CD56+ and CD19+CD45+ cells counts and percentages from baseline to 2-to-6 months after treatment; (2) the association of these phenotypes with 5 gradual categories of viral load; and (3) the accuracy of CD16+CD56+ and CD19+CD45+ cells counts in indicating AIDS stage defined as CD4+ < 200 cells/mm^3^. The second and third objectives were tested using a pooled analysis (N = 300–373). Result: The median CD19+CD45+ and CD16+CD56+ counts increased by 1.9-fold and 1.3-fold after treatment respectively (*p* < 0.001). A negative correlation of viral load with both CD16+CD56+ (ρ = −0.29, *p* < 0.001) and CD19+CD45+ (ρ = −0.34, *p* < 0.001) counts was observed. CD16+CD56+ count < 73 cells/mm^3^ and CD19+CD45+ count < 166.5 were indicative for AIDS with 95.5% and 63.6% sensitivity respectively. Conclusions: Findings advocate for the usefulness of CD16+CD56+ and CD19+CD45+ phenotyping in characterizing the severity of HIV infection and its impact on both the humoral and cellular immunity, as well as monitoring the effectiveness of treatment.

## 1. Introduction

In spite of the progress in antiretroviral therapy (ART) and the global efforts to improve the therapy strategies, nearly 2 million individuals are infected with human immunodeficiency virus (HIV) each year [1]. The effective monitoring of people living with HIV (PLHIV) provides crucial data of high clinical and epidemiological value. These data enable a real-time insight into disease activity, notably the progression to the acquired immunodeficiency syndrome (AIDS) stage, patients’ infectiveness, and the efficacy of ART, which also enables the diagnosis of eventual treatment failures [2]. Additionally, monitoring ART toxicity is recommended, as delaying drug replacements after developing adverse effects may not only harm patients but also lead to nonadherence resulting in drug resistance and treatment failure. This might compromise the available ART regimens, leading to increased HIV incidence, the spread of drug-resistant HIV, and increased morbidity and mortality [3]. Thus, besides measuring the HIV viral load in patients’ sera, CD4+ T lymphocytes count testing was developed as an indicator of the patients’ immune status. This test, called immunophenotyping, uses cytometers and cell sorters enabling the identification of different lymphocyte subsets using monoclonal antibodies. Early clinical usage of CD4 immunophenotyping established a correlation between CD4 count and AIDS stage. Thus, CD4 has ever since been widely used as a strong prognostic factor and a criterial term for the definition of the AIDS stage [4]. Furthermore, the CD4+:CD8+ ratio was also reported as a marker of a great clinical usefulness in HIV [5].

However, monitoring multiple cellular markers of immune activation may provide a more accurate evaluation of the immune status of the patient and enable a better understanding of the occurrence of opportunistic infections in PLHIV with normal or subnormal CD4 count. Among the immune functions that have been reportedly altered in HIV infection are naïve CCR7, CD27, and CD45RA T cells along with memory CD57, PD1, CD160, LAG-3, Tim-3, CTLA4, and 2B4 T cells, along with other naïve and memory B cells [6]. CD16 phenotype alteration has also been reported in HIV, and may be relevant in HIV monitoring and management [7]. The CD19+ phenotype alone has not been investigated yet as a marker in HIV monitoring. It was previously demonstrated that HIV infection involves CD19+CD45+ cells, which interact with HIV virions or viral proteins by binding via complement receptor CD21, a mechanism which enhances viral dissemination and accelerates B cell depletion by apoptosis [8].

This study aimed to assess the value of CD markers including CD16+CD56+ (NK cells) and CD19+CD45+ (B cells) in assessing and monitoring PLHIV, besides CD4 and the CD4/CD8 ratio. It tested three hypotheses: (1) the effect of treatment on the counts and percentages of the two CD markers; (2) the correlation of the two CD markers with HIV viral load; and (3) their performance in predicting AIDS status indicated by CD4 count < 200 cells/mm^3^.

## 2. Materials and Methods

### 2.1. Design and Participants

This was a retrospective study conducted at the Immunodiagnostic Department of King Abdulaziz University Hospital (KAUH), Jeddah, Saudi Arabia. It involved all PLHIV who were diagnosed, first treated, and followed up at the Infectious Disease Department of KAUH between 2009 and 2016. After exclusion of patients who had no relevant or follow-up data, a total of 82 PLHIV were included. The institutional review board of KAUH reviewed the study protocol and provided the ethical approval for the collection and use of the relevant data, under approval no. 197-22.

### 2.2. Data Collection

The researcher reviewed all laboratory records of the eligible participants and used an Excel sheet to collect baseline (at diagnosis) and all follow-up data of the following parameters:

– HIV RNA/mL plasma level at diagnosis, which was ranked into 5 levels: ≤500, 501–3000, 3001–10,000, 10,001–30,000, and >30,000 copies/mL. This ranking corresponds to 5 risk categories, determined by Mellors et al., and was demonstrated to be associated with differential 6-year risks of progression to AIDS of 5.4%, 16.6%, 31.7%, 55.2%, and 80.0% [9].
– Blood count data including total white blood cells (WBCs), lymphocyte count, platelets, and monocyte count.
– Counts and percentages of the following phenotypes: CD3+CD45+, CD3+CD4+CD45+, CD3+CD8+CD45+, CD16+CD56+ (NK cells), and CD19+CD45+ (B cells), and CD4:CD8 ratio.

Additionally, demographic data including age, gender, and nationality were collected.

### 2.3. Diagnostic Immunology Assays

#### 2.3.1. Immunophenotyping of Lymphocytes

Immunophenotyping of lymphocytes was carried out at our diagnostic immunology division according to the commercial BD kits protocol (BD multitest TM Kit; Becton and Dickinson, San Jose, CA, USA). Briefly, patients’ blood samples were collected in ethylenediaminetetraacetic acid (EDTA) anticoagulant test tubes (Vacutainer Tubes with K3EDTA-Becton Dickinson, Plymouth, UK) for immunophenotyping of lymphocyte profiles. As we used BD TrucontTM tubes, we did not use the live/dead stain to check the viability of the cells. The patients’ anti-coagulated whole blood was treated with BD CD3/CD8/CD45/CD4 and CD3/CD16+CD56/CD45/CD19 antibodies (mouse IgG1 heavy chains and kappa light chains and mouse IgG2b for the CD56 antibody). Subsequently, conjugates were added to pre-incubated patient samples, including CD45-PerCP, CD3-FITC, CD8-PE, and CD4-APC-A CD16+CD56-PE (BD Bioscience, San Jose, CA, USA).

**Figure 1:**
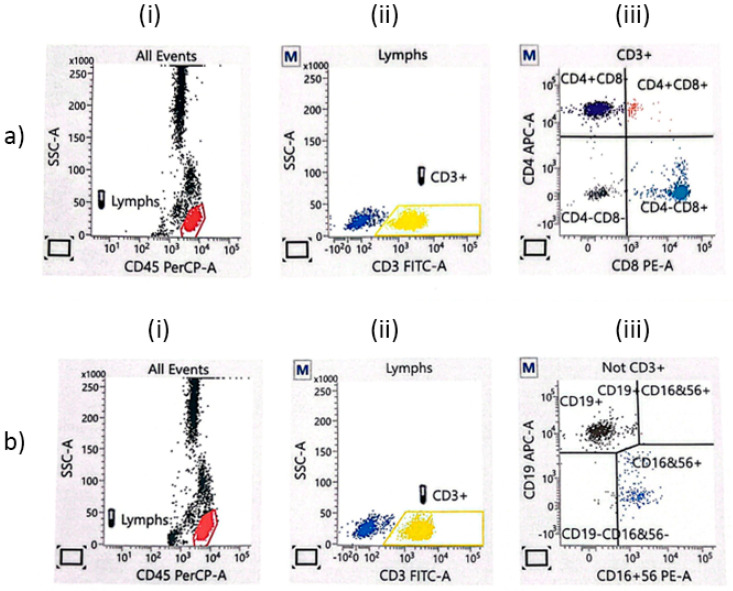
The flow cytometry gating strategy for TBNK lymphocytes. (**a**) CD3+CD4+ (APC-A) and CD3+CD8+ lymphocytes (PE-A): (**i**) all events; (**ii**) CD3+ T lymphocytes; (**iii**) the proportion of CD8+ cytotoxic T cells and CD4+ T helper cells. (**b**) CD19+ B lymphocytes (APC-A) and NK lymphocytes (PE-A): (**i**) all events; (**ii**) CD3− lymphocytes; (**iii**) the proportion of NK cells (CD16+CD56+) and B cells (CD19+).

After that, lymphocytes and lymphocyte sub-populations were determined by the flow cytometry method using BD FACSCalibur™ (Becton and Dickinson, San Jose, CA, USA). Briefly, in each sample, 10,000 cells were analyzed, and the results were expressed as the percentage (proportion) of the examined cells in each subpopulation of lymphocytes. Cell subpopulations were analyzed in BD program, version 6.1.3 (DB Biosci-ence, San Jose, CA, USA). In the analyzed samples, lymphocytes were defined via the gating strategy for TBNK (Figure 1). The absolute number of individual subpopulations (the number of cells per 1 mL of blood) was determined by the formula Abs CD4 (CD8) = WBC × Lym/100 × (% positive cells Cd4 or CD8)/100.

During analysis, the absolute number (cells/µL) of positive cells in the sample was determined by comparing cellular events to bead events. In the study setting, we used BD Multiset software to determine the absolute count automatically, which can be obtained using the following equation:

$$A=\frac{X}{Y}\times \frac{N}{V}$$

where *A* is the absolute count of the cell population; *X* is the number of positive cell events; *Y* is the number of bead events; *N* is the number of beads per test, which is found on the BD Trucount foil pouch and can vary from lot to lot; and *V* is the test volume. In addition, absolute counts were compared to the CBC results obtained for each patient using the hematology analyzer.

#### 2.3.2. Nucleic Acid Amplification Test for the Quantitation of Human HIV-1 Ribonucleic Acid (RNA) in Human Plasma

The commercial COBAS^®^ AmpliPrep/COBAS^®^ TaqMan^®^ HIV-1 Test, v2.0 (Roche, Switzerland) using Polymerase Chain Reaction (PCR) technology was used to achieve maximum sensitivity and dynamic range for the quantitative detection of HIV-1 group M and group O RNA in EDTA anti-coagulated plasma. The HIV-1 RNA signal was calculated in the specimens and controls by comparing their concentrations to the quantitation standard concentrations in each specimen and control. The test range was 20–10,000,000 copies/mL.

### 2.4. Statistical Approaches

The researcher used three different analytical approaches to test the 3 hypotheses, using two different datasets: a paired dataset and a pooled dataset.

#### 2.4.1. Hypothesis 1

The first analytical approach consisted of a paired analysis testing the change, from baseline (pre-ART) to follow-up (post ART) times, in NK cell and B cell counts along with HIV RNA/mL plasma levels and the other tested parameters. The follow-up time considered for this analysis was 2 to 6 months, depending on the patient, corresponding to the initial assessment of ART therapeutic response. Thus, the first approach tested the effect of treatment on NK cells and B cell counts and percentages, as well as on the levels of HIV RNA/mL plasma and the other phenotypes. It was hypothesized that a decrease in HIV RNA/mL plasma levels would be concomitant with an increase in NK cells and B cells and other phenotypes. Such an increase would support the relevance of using the given parameter as an indicator for a complete response to ART.

#### 2.4.2. Hypothesis 2

The second analytical approach tested the bivariate correlation of NK cell and B cell counts and percentages with HIV RNA/mL plasma levels. We hypothesized that a negative relationship between HIV RNA/mL plasma levels and a given phenotype (NK cells or B cells) would indicate the value of this phenotype in monitoring PLHIV and their response to antiretroviral treatment. This analysis used the pooled data of all patients at all follow-up times, subject to availability of the relevant data.

#### 2.4.3. Hypothesis 3

The third hypothesis consisted of analyzing the sensitivity of NK cell and B cell counts in indicating AIDS status, defined by CD4 cell count < 200 cells/mm^3^ [10]. This analysis used the pooled database.

### 2.5. Statistical Methods

Both databases were edited, processed, and analyzed using the Statistical Package of Social Science (SPSS), version 21, for Windows (IBM, Chicago, IL, USA). Descriptive statistics were used for baseline data to present means and standard deviations (SD)s, medians and interquartile ranges (IQRs), or frequencies and percentages, as applicable.

To test the first hypothesis, the related-samples Wilcoxon signed-rank test was used to analyze the paired change in HIV RNA/mL plasma rank and NK cell and B cell counts and percentages, in addition to the other parameters including total WBCs; lymphocyte count; platelets; monocyte count; counts and percentages of CD3+CD45+, CD3+CD4+CD45+, CD3+CD8+CD45+; and CD4:CD8 ratio. The results are presented as medians with IQR and the *p*-value.

In the second analytical approach, using pooled data, Spearman’s coefficient (ρ) was used to analyze the bivariate correlation of HIV RNA/mL plasma level with NK cell and B cell counts and percentages as well as with the other parameters. Additionally, the Kruskal–Wallis test was used to analyze the variance in the different phenotypes by HIV RNA/mL plasma rank (≤500, 501–3000, 3001–10,000, 10,001–30,000, and >30,000 copies/mL); the results are presented as the median and IQR of the given parameter in the given HIV RNA/mL plasma rank.

To test the third hypothesis, Receiver Operating Characteristics (ROC) curve analysis was carried out to analyze the accuracy of NK cell and B cell counts as well as CD3+ count in indicating AIDS status, with calculation of the area under the curve (AUC). The ROC curve analysis was completed by the determination of the best cutoff of each parameter and the related sensitivity and specificity by applying the Youden’s index method to the ROC curve coordinates.

A *p*-value < 0.05 was considered to reject the null hypothesis.

## 3. Results

### 3.1. Baseline Characteristics

**Figure 2:**
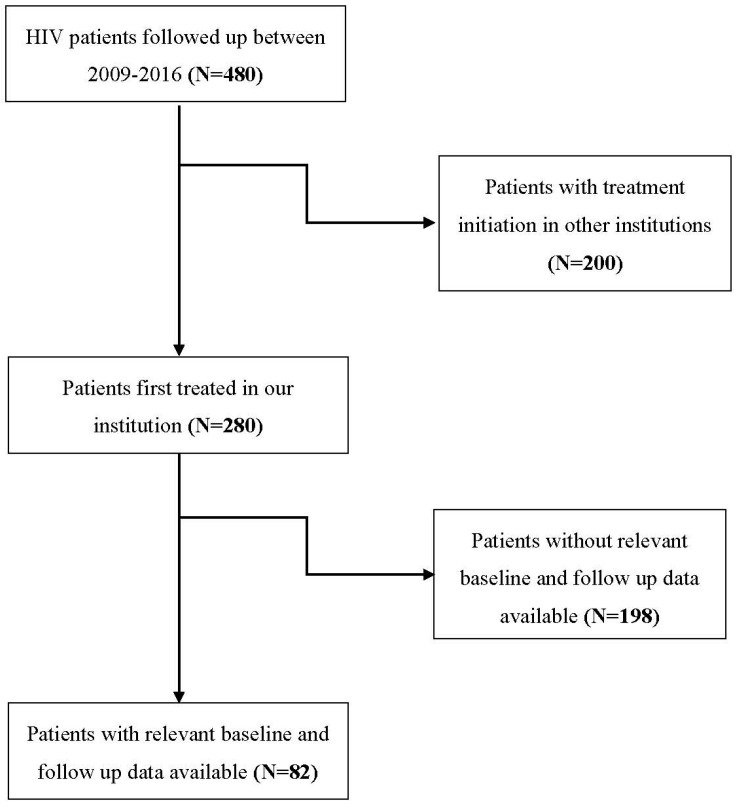
Flowchart of the participants.

The flowchart of participants is depicted in Figure 2. Eighty-two PLHIV were included in the study, forty-four (53.7%) were male and the mean (SD) age was 49.00 (13.83) years. At diagnosis, the majority of patients (64.6%) had very high HIV RNA/mL plasma titers (>30,000 copies/mL). HCV and HbsAg were negative in all tested patients. Other serology findings and mean (SD) values of blood count parameters and immunophenotyping are presented in Table 1.

### 3.2. Change in HIV RNA/mL Plasma from Baseline to Outcome

There was a remarkable decrease in HIV RNA/mL plasma titers from the baseline to endpoint, with 61 (74.4%) of the patients having ≤500 copies/mL, while only 11 (13.4%) had more than 10 000 copies/mL (Table 2). The mean (SD) follow-up time from baseline to last follow-up was 30.8 (26.9) weeks and the median (IQR) was 22 (20) weeks (the results are not presented in tables).

### 3.3. Change in Blood Count and Phenotyping Parameters

From baseline to follow-up, the median NK cell and B lymphocyte counts increased 1.9- and 1.4-fold, respectively (*p* < 0.001). Additionally, there was a significant increase in total lymphocyte count (*p* < 0.001) as well as in the counts of other phenotypes including CD3+CD45+ (*p* < 0.001), CD3+CD4+CD45+ (*p* < 0.001), and CD3+CD8+CD45+ (*p* < 0.001). Furthermore, both CD3+CD4+CD45+ percentage (*p* < 0.001) and CD4/CD8 ratio (*p* = 0.001) increased significantly (Table 3).

### 3.4. Correlation of Immunophenotyping Parameters with HIV RNA/mL Plasma Levels

By considering pre-treatment findings only (N = 82), both NK cells (ρ = −0.26) and B cells (ρ = −0.18) were inversely correlated with HIV RNA/mL plasma; however, this was statistically significant for NK cells but not for B cells (*p* = 0.144). In the post-treatment period (N = 82), neither NK cells (ρ = −0.15, *p* = 0.187) nor B cells (ρ = −0.12, *p* = 0.283) showed a significant correlation with HIV RNA/mL plasma (Table 4). It should be noted that the latter correlation analyses are limited by the low sample size (N = 82) and the skewed distribution of the participants within the HIV RNA/mL plasma categories both in the pre- and post-treatment periods, as shown in Table 2.

By pooling baseline and different follow-up data of all patients, 300 to 373 observations were obtained, depending on the parameter. Bivariate correlation analysis showed that HIV RNA/mL plasma titer was negatively correlated with counts of all the studied lymphocyte phenotypes (*p* < 0.05); however, the relationship was weak to moderate, with Spearman’s coefficient ρ ranging between −0.17 and −0.38. The relationship of HIV RNA/mL plasma was relatively stronger with CD3+CD4+CD45+ count (ρ = −0.38, *p* < 0.001) and B cell count (ρ = −0.34, *p* < 0.001) (Table 5).

### 3.5. Significance of NK Cell Count, B Cell Count, and CD3+ Count in Indicating AIDS Status (Pooled Data)

**Figure 3:**
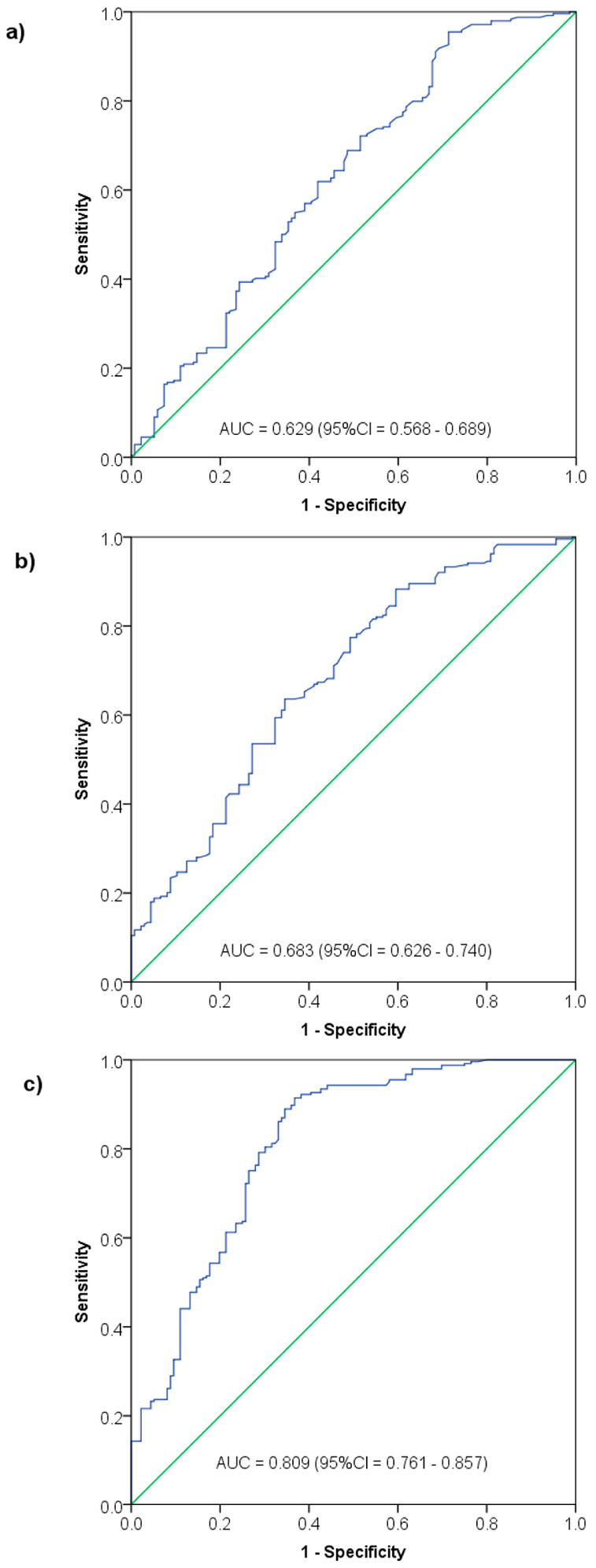
Significance of NK cell count (**a**), B cell count (**b**) and CD3+ cell count (**c**) in indicating AIDS status among HIV patients—Receiver Operating Characteristics (ROC) curve analysis.

The ROC curve analysis showed NK cell (AUC = 0.629, *p* < 0.0001) and B cell (AUC = 0.683, *p* < 0.0001) counts to be equally indicative of AIDS status, while CD3+ count had a higher AUC of 0.809 (*p* < 0.0001) (Figure 3). By applying Youden’s index, an NK cell count < 73 cells/mm^3^ was indicative for AIDS status with 95.5% sensitivity and 28.7% specificity, and a B cell count < 166.5 cells/mm^3^ was indicative for AIDS with 63.6% sensitivity and 65.4% specificity. By contrast, a CD3 count < 876.5 cells/mm^3^ was indicative for AIDS with 91.4% sensitivity and 63.2% specificity (Table 6).

## 4. Discussion

### 4.1. Summary of Findings

This retrospective laboratory-based study explored the significance of NK cells and B cells in monitoring newly diagnosed and treated PLHIV in a referral center in Western Saudi Arabia. It used two different databases to analyze the effect of ART response on NK cell and B cell counts, the correlation of NK cell and B cell counts and percentages with viral load, and the sensitivity of NK cell and B cells count in indicating AIDS status. A paired pre-to-post-treatment approach demonstrated a significant increase in NK cell and B cell counts, as well as CD4+ and overall CD3+ counts, as a result of ART initiation, occurring after a median follow-up period of 22 weeks. A pooled database was used to demonstrate both NK cell and B cell counts to be negatively correlated with HIV RNA/mL plasma levels, with correlation coefficients comparable to those found with CD4+ and CD3+ T cells. Additionally, NK cell and B cells count were demonstrated to be significantly predictive for AIDS status, with a sensitivity as high as 95.5% for an NK cell count < 73 cells/mm^3^.

### 4.2. Discussing Baseline Observations

It is of great importance to note that the majority (65%) of the patients had high HIV RNA/mL plasma levels at diagnosis and almost half of them (48.8%) were diagnosed at the AIDS stage. This is indicative of a significant delay in the diagnosis of HIV in Saudi Arabia, which should be considered a high-priority public health issue by the health authorities. Late HIV diagnosis not only impacts the health outcome and life expectancy of the infected individuals [11], but also increases the risk of virus dissemination in the community [12], ultimately increasing the burden of the disease. Therefore, several models have been proposed to characterize the delay in HIV diagnosis, and most of these agreed on the reliability of CD4 count in defining the late presentation, notably the concurrent diagnosis of HIV and AIDS [13]. Furthermore, the consequence of late HIV diagnosis is more detrimental in cases of inaccessible, delayed or inadequately conducted treatment. In a large Chinese cohort study of 528,234 patients newly diagnosed with HIV, where 34% had a late diagnosis, the one-month mortality rate among individuals who were diagnosed with a CD4 count < 50 cells/mm^3^ was 16% in the absence of ART, in comparison to <1% in whom ART was appropriately conducted. The one-year mortality rate was 64% versus 13.3% in the two groups, respectively [14]. Altogether, these observations highlight the urgent need to enhance routine testing in Saudi Arabia to reduce late HIV diagnosis and promote early initiation of ART.

### 4.3. Impact of HIV Infection on NK Cells

The findings from the present study support the reliability of using NK cells as a biomarker of the severity of HIV infection and the therapeutic response to ART among patients newly diagnosed with HIV. Both the count and percentage of NK cells were negatively correlated with HIV viral load and increased in response to ART initiation. Furthermore, an NK cell count < 73 cells/mm^3^ was indicative for AIDS status with 95.5% sensitivity. NK cells are innate lymphoid cells that ensure nonspecific immune response against tumoral and virus-infected cells [15], and contribute to the regulation of adaptive immunity [16,17]. The impact of HIV infection on this cell population has already been demonstrated. The activity of NK cells is significantly altered among PLHIV [18], and several theories have been elaborated to explain such an alteration [19,20,21]. It was even proposed that NK cell activity could be a prognostic indicator for the progression to the AIDS stage [22], which is consistent with our findings showing an association between NK cell count and AIDS status. In 2009, Mansour et al. observed a significant decline in NK cells and other CD3− phenotypes in all-stage PLHIV in comparison to non-HIV controls. By stratifying HIV into three severity stages, patients at the AIDS stage had a significantly lower NK cell count (mean ± SEM = 121 ± 11 cells/mm^3^) compared with asymptomatic patients (172 ± 15) and those with generalized adenopathy (187 ± 24) [23]. Other data by Vuillier et al. found a significant decrease in both NK and B cells, and the authors suggested that such a depletion “accounts for the decline in low-density CD8+ lymphocytes in HIV positive group” [24]. Interestingly, a recent review characterized the pathogenic impact of HIV infection on the frequency and functions of NK cells, which impairs their anti-viral activity notably by the upregulation of inhibitory NK cell receptors (iNKRs) and downregulation of activating NK cell receptors (aNKRs), both of which are sensitive to the viral load. On the other hand, the same review demonstrated the reversibility of these pathological changes after a well-conducted ART [25]. These observations are consistent with findings from the present study showing a negative correlation between NK cell count and viral load as well as a significant increase in NK count after treatment.

Moreover, it has been demonstrated that CMV infection induces adaptive changes to NK cells, resulting in the emergences of certain subsets such as an increase in the number of mature CD56dimNKG2A+CD57+ NK cells. This is associated with alterations in the signaling and cytokine responsiveness of NK cells [26,27]. However, in the present study, only one participant had an active CMV infection, indicated by positive IgM, which would not impact the cohort findings.

### 4.4. Impact of HIV Infection on CD19+ Lymphocytes

In the present study, the frequency of CD19+ lymphocytes was demonstrated to be significantly lower among newly diagnosed HIV patients before treatment, which increased after initiation of ART. Furthermore, the B cell count was negatively correlated with the viral load; however, the best B cell count cutoff was not sensitive (63.6%) or specific in indicating AIDS status. CD19+ lymphocytes encompass several subsets and have an important role, notably in T cell activation [28]. The effect of HIV infection and the plasma viremia on CD19+ and other subsets of B lymphocytes have been less characterized than the effect on CD4+ and CD8+ T cells in the early years of the HIV epidemic. However, several pathogenic mechanisms involving B cells have been discovered, contributing to the understanding of the impaired humoral immunity and its association with the development of opportunistic infections among PLHIV [29,30,31]. Clinically, Samuelsson et al. observed high levels of CD19+ cell apoptosis among PLHIV with rapidly progressing disease, which was correlated with viral load and the level of CD4+ cell apoptosis. On the other hand, the authors observed a low rate of apoptosis in CD19+ cells in slowly progressing forms of HIV [32]. These observations are consistent with our findings and support the relevance of monitoring CD19+ cells as an indicator for the impact of HIV infection on humoral immunity. Other data demonstrated other B lymphocyte subsets to be prone to apoptosis in PLHIV, such as CD10+ cells, and that there are different mechanisms of apoptosis [33]. Other observations by Moir et al. showed a generalized impairment of B lymphocyte functions in association with high HIV viral load, notably the abnormal proliferation of specific subsets of B lymphocytes, such as CD21+ cells, that are responsible for hypergammaglobulinemia [34]. Another study by Moir et al. showed a significant increase in and normalization of CD19+ cell count after a twelve-month ART, which was concomitant with the increase in CD4+ T cells and decrease in HIV viral load [35]. This is consistent with our findings and supports the responsiveness of CD19+ cells to ART and its reliability as an indicator for ART effectiveness.

### 4.5. Clinical Implications

This study provides insights into the role of NK cells and B cells in monitoring PLHIV, especially those newly diagnosed, which may have various clinical implications:

– One of the potential applications is in the comprehensive assessment of immune status in HIV-infected individuals, especially at diagnosis.
– Clinicians can further use these CD markers to track the effectiveness of ART and make informed decisions regarding treatment adjustments when necessary. This constitutes an area for research into the added value of monitoring these parameters for more effective management.
– More notably, NK cell count has shown remarkable sensitivity in indicating AIDS status, with 95.5% sensitivity at a cutoff of <73 cells/mm^3^. The analysis of the kinetics of NK count changes during HIV infection would present a potential application in the early identification of patients progressing towards AIDS, allowing for timely intervention.
– On the other hand, the inclusion of these markers in routine assessment and monitoring will entail extra costs that may require further evidence to support their added clinical value and cost-effectiveness. The complexity of the assay and the need for specialized equipment may limit its accessibility, especially in resource-limited settings where the burden of HIV is often the highest. It is to note that, in our department, the phenotyping assay systematically analyzes all lymphocyte subsets, including CD4, CD8, and other CD markers.
– Furthermore, the effective implementation of these markers requires technical expertise not only in performing the assay but also in interpreting the results, which may not be readily available in all clinical settings.
– The alarming rates of late HIV diagnosis, with a substantial portion of patients already in the AIDS stage at the time of diagnosis, underline the need for increased awareness and routine testing in Saudi Arabia. Early identification of HIV infection is paramount in preventing the progression to AIDS and reducing the transmission of the virus within the community.

### 4.6. Research Implications

The findings from this study provide various research perspectives, among which are the following:

– Further research is warranted to establish the correlations between viral load and NK cell as well as B cell counts, unlike CD4+ cell counts, which have a well-established threshold for staging HIV infection. Defining standard thresholds is crucial for streamlining clinical interpretation and ensuring consistent results across different laboratories and technicians.
– Secondly, the longitudinal stability of the markers in question needs to be evaluated. CD4+ cell counts are known to fluctuate with disease progression and treatment, and it is necessary to understand how NK and B cell counts may vary over time and in response to ART.
– The markers’ specificity and sensitivity in detecting various stages of HIV across different populations need confirmation to ensure accurate disease staging, without interfering with other conditions.
– The clinical benefits of these markers and their cost-effectiveness still need to be proven through rigorous trials before they can be adopted into standard HIV management protocols.

### 4.7. Limitations

The present study is limited by the retrospective design, which resulted in an unavailability of relevant data in a number of patients. Additionally, the availability of CD16+CD56+ and CD19+ phenotyping data may be contingent upon logistical and technical resources throughout the study period, potentially introducing a selection bias. Finally, the internal validity of the findings may be compromised by the use of pooled data, which may explain both the low AUC in the ROC curve results and the relatively low levels of correlation between HIV RNA/mL plasma and NK and B cells. This limitation stems from the heterogeneity of the pooled data that combined pre- and post-treatment results, thereby not accounting for the complex immunological changes that may be triggered by ART and that may interfere with the levels of the different phenotypes. Nevertheless, the pooled analysis was carried out to test whether the changes in NK and B cell counts are concomitant with the changes in CD4 and HIV RNA/mL plasma levels, and thus could reflect the immune status of the patients regardless of the treatment status and other immunological mechanisms. Still, further evidence is warranted from adequately sized, prospective paired data using fixed intervals to characterize the proportional change over time of CD markers in correlation with the change in HIV RNA/mL plasma levels after treatment initiation.

## 5. Conclusions

The present analysis showed that the frequencies of NK cells and B cells were significantly impacted in patients newly diagnosed with HIV proportional with the severity of the disease indicated by a high viral load and low CD4+ count. Moreover, the NK cell count was highly sensitive in indicating AIDS stage defined as a CD4+ count < 200 cells/mm^3^, while B cells showed a low sensitivity. Additionally, NK cell and B cell counts were responsive to the treatment and were significantly increased after the initiation of ART. This advocates for the usefulness of comprehensive lymphocytes phenotyping in characterizing the severity of HIV infection and its impact on both innate and adaptive immunity, as well as in monitoring the effectiveness of ART. However, further prospective studies are warranted to explore the cost-effectiveness and long-term relevance of these biomarkers in chronic HIV. Moreover, we noted an alarming proportion of patients with HIV diagnosed at the AIDS stage or with high viral loads, which requires urgent measures.

## Figures and Tables

**Table 1:** Baseline demographic and laboratory characteristics (N = 82).

Parameter	Category	Mean	SD	N	%
Age (N = 82)	Years	49.0	13.83		
Gender (M = 82)	Male			44	53.7
	Female			38	46.3
Nationality (N = 82)	Saudi			34	41.5
	Yemeni			28	34.1
	Other			20	24.4
HCV serology (N = 82)	Negative			82	100.0
	Positive			0	0.0
HbsAg (N = 81)	Negative			81	98.8
	Positive			0	0.0
Toxoplasmosis IgM (N = 31)	Negative			31	37.8
	Positive			0.0	0.0
CMV IgG (N = 65)	Negative			2	2.4
	Positive			63	76.8
CMV IgM (N = 6)	Negative			5	6.1
	Positive			1	1.2
AIDS (CD4 < 200 cells/mm^3^) (N = 80)	No			41	51.3
	Yes			39	48.8

Due to missing data for some patients, the values for certain variables do not sum to the total of 82.

**Table 2:** Progression of HIV RNA/mL plasma from baseline to outcome.

Viral Load (HIV RNA/mL Plasma)	Baseline		After Treatment		Statistics * (*p*-Value)
	N	%	N	%	
≤500	13	15.9	61	74.4	5.00 (<0.0001)
501–3000	4	4.9	8	9.8	
3001–10 000	7	8.5	2	2.4	
10.001–30,000	5	6.1	4	4.9	
>30,000	53	64.6	7	8.5	

* Related-samples Wilcoxon signed-rank test.

**Table 3:** Change in phenotype parameters (nonparametric tests).

Parameter	N	Baseline			After Treatment			Change	*p*-Value
		Median	IQR (Q1, Q3)		Median	IQR (Q1, Q3)			
Total WBC	78	4.27	3.52	7.28	4.26	3.72	6.45		0.911
Lymphocyte count	69	1.54	0.94	3.54	2.22	1.51	3.58	⬀	<0.001 *
Platelets	80	227	191.0	340.0	254	224.00	362.00		0.459
Monocyte count	69	0.57	0.38	1.46	0.52	0.35	0.87		0.177
CD3+CD45+ count	82	908	479	1903	1630	845	2638	⬀	<0.001 *
CD3+CD45+ Percentage	82	75	66.00	83.75	76	71.00	80.00		0.229
CD3+CD4+CD45+ count	80	203	29	486	311	146	613	⬀	<0.001 *
CD3+CD4+CD45+ %	80	15	4.0	25.0	14	7.00	30.00	⬂	<0.001 *
CD3+CD8+CD45+ count	63	556	277	1276	979	427	1579	⬀	<0.001 *
CD3+CD8+CD45+ %	63	53	34.25	66.00	48	35.00	65.00		0.193
CD16+CD56+ (NK) count	82	135	70	237	258	102	360	⬀	<0.001 *
CD16+CD56+ (NK) %	82	11	8.00	14.00	10	8.00	15.00		0.453
CD19+CD45+ (B cells) count	80	111	60	201	150	101	390	⬀	<0.001 *
CD19+CD45+ (B cells) %	80	10	6.00	15.00	9	5.00	15.00		0.858
CD4/CD8 ratio	59	0.29	0.06	0.67	0.37	0.14	0.64	⬀	0.001 *

N: number of valid data entries. Test: related-samples Wilcoxon signed-rank test; IQR: interquartile range; arrows indicate rise (⬀) or drop (⬂) of the given parameter from baseline to post-treatment times; * statistically significant result (*p* < 0.05).

**Table 4:** Correlation of immunophenotyping parameters with HIV RNA/mL plasma levels at baseline and outcome (N = 82).

Time	Parameter	Viral Load (HIV RNA/mL Plasma)		NK Cells		B Lymphocytes	
		ρ	*p*-Value	ρ	*p*-Value	ρ	*p*-Value
Pre-treatment	Viral Load	-	-	−0.26	0.020 *	−0.18	0.114
	CD16+CD56+	−0.26	0.020*	-	-	0.58	<0.001 *
	CD19+CD45+	−0.18	0.114	0.58	<0.001 *	-	-
Post-treatment	Viral Load	-	-	−0.15	0.187	−0.12	0.283
	CD16+CD56+	−0.15	0.187	-	-	0.46	<0.001 *
	CD19+CD45+	−0.12	0.283	0.46	<0.001 *	-	-

ρ: Spearman’s rho; viral load: HIV RNA/mL plasma; * statistically significant result (*p* < 0.05).

**Table 5:** Correlation of phenotyping parameters with HIV RNA/mL plasma (pooled data).

Parameter	≤500		501–3000		3001–10,000		10,001–30,000		>30,000		Kruskal–Wallis Test		Bivariate Correlation	
	Median	IQR	Median	IQR	Median	IQR	Median	IQR	Median	IQR	Statistics	*p*-Value	Spearman’s Rho	*p*-Value
Total WBC	4.8	2.52	4.45	1.92	4.49	1.23	4.53	2.81	3.94	3.13	8.65	0.070	−0.15	0.005 *
Lymphocyte count	1.97	2	1.54	3.15	2.29	11.1	2.33	1.44	1.54	8.52	3.75	0.441	−0.05	0.379
Platelets	272	110	331	139	259	38.8	226	14	227	113	29.43	<0.001 *	−0.21	<0.001 *
Monocyte count	0.52	0.59	0.54	0.77	0.56	5.34	0.57	0.32	0.58	6.5	0.52	0.971	0.04	0.524
CD3+CD45+ count	1564	1450	1185	1684	1055	926	1287	585	867	980	41.07	<0.001 *	−0.32	<0.001 *
CD3+CD45+ percentage	75.5	15	76	10	74	3	79	0.75	74	16	4.28	0.369	−0.18	0.731
CD3+CD4+CD45+ count	415	505	167	184	432	402	412	155	110	255	56.04	<0.001 *	−0.38	<0.001 *
CD3+CD4+CD45+ %	19.5	24.3	12	15	30	16	24	9.75	7	17	38.76	<0.001 *	−0.27	<0.001 *
CD3+CD8+CD45+ count	893	885	711	1110	535	521	871	591	556	910	14.17	0.007 *	−0.17	0.002 *
CD3+CD8+CD45+ %	45	30.8	64	23	43	14.3	49	5	61	22.5	23.83	<0.001 *	0.22	<0.001 *
CD16+CD56+ (NK) count	246	264	186	238	83	90	150	145	128	148	36.52	<0.001 *	−0.29	<0.001 *
CD16+CD56+ (NK) %	11	8.25	9	4	9.5	6	9	4.5	13	8	5.89	0.208	0.02	0.776
CD19+CD45+ (B cells) count	209	259	163	139	159	180	129	226	105	128	41.81	<0.001 *	−0.34	<0.001 *
CD19+CD45+ (B cells) %	10	10	8	11	14	10.3	11	4.75	10	8.5	2.21	0.698	−0.06	0.266
CD4/CD8 ratio	0.47	0.84	0.14	0.307	0.79	0.12	0.47	0.25	0.12	0.278	48.76	<0.001 *	−0.35	<0.001 *

IQR: interquartile range; * statistically significant result (*p* < 0.05). The bakcground color for the last two columns marks a separation from the other statistics.

**Table 6:** Diagnostic accuracy of NK and B cell levels in indicating AIDS status among HIV patients.

Parameter	AUC (95%CI)	SE	*p*-Value	Youden’s Index	Cutoff Value	Sensitivity	Specificity
NK cell count	0.629 (0.568, 0.689)	0.031	<0.0001	0.242	<73 cells/mm^3^	95.5%	28.7%
B cell count	0.683 (0.626, 0.740)	0.029	<0.0001	0.290	<166.5 cells/mm^3^	63.6%	65.4%
CD3+ count	0.809 (0.761, 0.857)	0.024	<0.0001	0.547	<876.5 cells/mm^3^	91.4%	63.2%

AUC: area under the corresponding Receiver Operating Characteristics (ROC) curve; SE: standard error.

## Data Availability

The datasets generated and analyzed during the current study are not publicly available due to privacy and ethical considerations. However, they are available from the corresponding author upon reasonable request via email.
